# Astrocytes as Active Participants in Memory

**DOI:** 10.1111/jnc.70258

**Published:** 2025-10-14

**Authors:** Shay Meron Asher, Inbal Goshen

**Affiliations:** ^1^ Edmond and Lily Safra Center for Brain Sciences (ELSC) The Hebrew University of Jerusalem Jerusalem Israel

**Keywords:** astrocyte, chemogenetics, engrams, memory, optogenetics, plasticity

## Abstract

Recent research has revealed the crucial role of astrocytes in memory processes, challenging the traditional view of exclusively neuronal‐based cognitive functions. This review summarizes current findings on astrocytic involvement in memory, focusing on their interactions with neurotransmitters, various biomolecules, and neurons in different brain regions. We include studies that discuss how astrocytes modulate synaptic transmission and contribute to plasticity, thereby influencing memory formation, consolidation, and recall. We conclude by providing an overview of advanced techniques such as optogenetics, chemogenetics, calcium imaging, and engram research, which have provided new insights into the function of astrocytes and their role in memory processes.

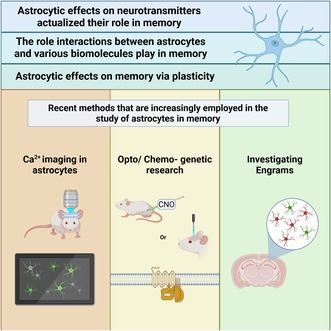

## Graphical abstract

This review explores how astrocytes support memory by modulating neurotransmitters, interacting with biomolecules, and taking part in synaptic plasticity. We discuss their role across various brain regions and spotlight cutting‐edge methods—such as Ca^2+^ imaging, optogenetics, chemogenetics, and engram analysis—that have revolutionized our ability to investigate astrocytic function in memory. Together, the findings summarized in this review underscore the growing recognition of astrocytes as central to cognitive processes.

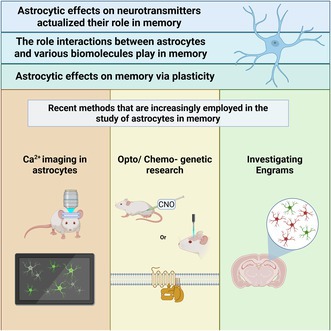

**Abbreviations**
3‐NT: 3‐nitrotyrosine
A1Rs: A_1_ receptors
ACC: anterior cingulate cortex
AMP: adenosine monophosphate
ATP: adenosine triphosphate
B3HB: β‐hydroxybutyrate
BLA: basolateral amygdala
Ca^2+^
: calcium
CeA: central amygdala
ChR2: channelrhodopsin‐2
CNiFERs: Cell‐Based Neurotransmitter Fluorescent Engineered Reporters
CNO: clozapine N‐oxide
CPA: conditioned place avoidance
CPP: conditioned place preference
CREB: cAMP Response Element‐Binding Protein
CRISPR: Clustered Regularly Interspaced Short Palindromic Repeats
DG: dentate gyrus
DREADDs: Designer Receptor Exclusively Activated by Designer Drug
FC: fear conditioning
fEPSPs: field excitatory postsynaptic potentials
GABA: ɣ‐aminobutyric acid
GABA_B_Rs: GABA_B_ receptors
GPCRs: G‐protein coupled receptors
GPR30: G‐protein coupled receptor 30
GRs: Glucocorticoid receptors
GTPase: guanosine triphosphate hydrolase
IGF1Rs: insulin‐like growth factor 1 receptors
iGluSnFr: intensity‐based glutamate‐sensing fluorescent reporter
IP3: Inositol 1, 4, 5‐triphosphate
IP3R2: IP3 receptor type 2
iβARK: 122‐residue inhibitory peptide from β‐adrenergic receptor kinase 1
KO: knock out
LC: locus coeruleus
LTP: long‐term potentiation
MCTs: monocarboxylate transporters
mEPSCs: miniature excitatory postsynaptic currents
METH: methamphetamine
mGluR: metabotropic glutamate receptors
MLC: megalencephalic Leukoencephalopathy with subcortical Cysts
MLC1: membrane protein MLC1
mPFC: medial prefrontal cortex
NAc: nucleus accumbence
nAChRs: nicotinic acetylcholine receptors
NE: norepinephrine
NFIA: transcription factor nuclear factor I‐A
NMDARs: N‐Methyl‐D‐aspartate receptors
NOL: novel object location
NOR: novel object recognition
OF: open field
PAPs: peripheral astrocytic processes
PJA: Praja1
RAC1: Ras‐related C3 botulinum toxin substrate 1
RAWM: radial arm water maze
SKG1: serum and glucocorticoid‐regulated kinase‐1
SOD2: superoxide dismutase 2
α_1_ARs: α_1_‐adrenergic receptors
β_2_ARs: β_2_‐adrenergic receptors

## 1 Introduction

Memory, the ability to encode, store, and retrieve information, is a fundamental cognitive process essential for adaptation and survival. Memory research has traditionally focused primarily on neurons, their connections, and the molecular mechanisms underlying synaptic plasticity. Although astrocytes are abundant cells in the central nervous system, they were long considered to be mere support cells, providing metabolic assistance and maintaining homeostasis in the brain. However, evidence shows that astrocytes are active participants in information processing and play crucial roles in modulating synaptic transmission, plasticity, and ultimately, memory formation and consolidation.

This review aims to provide an overview of research from recent years discussing the diverse roles of astrocytes in memory processes. We summarize studies that explored the complex interactions between astrocytes and different neurotransmitters and the contribution of astrocyte‐dependent metabolites to memory. The studies covered in this review emphasize the impact of manipulating specific astrocytic proteins and signaling pathways on memory performance, as well as the structural plasticity of astrocytes.

We also discuss recent technological advancements which have greatly enhanced our ability to study astrocyte function in vivo, such as astrocytic calcium (Ca^2+^) imaging during memory‐related functions, optogenetics and chemogenetics, and the tagging of astrocytes in memory engrams. These tools have provided unprecedented insights into the signaling capabilities of astrocytes, revealing their active participation in neural circuits underlying memory, and opening new opportunities for progress in our research field.

## 2 Astrocytic Effects on Neurotransmitters Actualize Their Role in Memory

Recent studies have shed light on how astrocytes detect, respond to, and regulate neurotransmitter signaling in a region‐specific manner, shaping memory processes across the brain. In the hippocampus, a key structure in memory formation, astrocytes actively participate in synaptic transmission by sensing neurotransmitters and modulating neuronal activity through gliotransmitter release. Hippocampal neurons in the CA1 region receive one of their main inputs from glutamatergic CA3 neurons via the Schaffer Collateral pathway and are modulated, for example, by norepinephrine (NE) inputs from the locus coeruleus (LC). These inputs stimulate astrocytic Ca^2+^ activity, leading to the release of glutamate and D‐serine (an NMDA receptor co‐agonist) from astrocytes in this area. These two molecules are of particular interest as glutamate serves as the primary excitatory neurotransmitter and NMDA receptors are critically involved in synaptic plasticity and memory formation (see ‘Astrocytic effects on memory via plasticity’ section). In addition, astrocytic D‐serine has been shown in the past to be required for supporting hippocampal‐prefrontal theta synchronization which is mandatory for intact memory (Sardinha et al. 2017) and to regulate NMDARs activity in CA3 → CA1 synapses which subsequently affects novel object recognition (NOR) memory (Langlais et al. 2025) or contextual fear conditioning (Papouin et al. 2017). This astrocytic release of glutamate and D‐serine is triggered by the activation of astrocytic α1‐adrenergic receptors (α_1_ARs) and affects NMDAR tone and memory formation (Koh et al. 2022). A knockout (KO) of a gene is a manipulation performed to prevent it from being expressed. D‐serine regulation mediates the effects of the KO of CB1 receptors in astrocytes, which impairs NOR memory (Robin et al. 2018). D‐serine levels can also be affected by the presence of SOD2, which is closely related to neurodegeneration in Alzheimer's disease and other memory‐related diseases (Baier et al. 2022). Knocking out SOD2 in hippocampal astrocytes leads to decreased D‐serine levels in both male and female mice. With that, female KOs show increased amounts of the rate‐limiting enzyme in D‐serine biosynthesis serine racemase (SRR), while in the male KOs, there is a decrease accompanied by a decreased learning index, decreased cognitive flexibility, and an increased amount of wrong choices in the radial arm water maze (RAWM), that is not observed in the female group (Baier et al. 2022).

In the dentate gyrus (DG) of the hippocampus, astrocytic connexin30, a gap junction‐forming protein, plays a role in modulating synaptic transmission and the formation of hippocampus‐dependent contextual memory. NE afferent signals from the LC have been shown to be relevant to learning, task execution, and performance improvement as a part of prediction error in rodents (Breton‐Provencher et al. 2022; Drummond et al. 2024) and in zebrafish (Mu et al. 2019), where it was also demonstrated that brainstem radial astrocytes exhibit calcium accumulation proportional to the number of failed swim attempts, represented by the NE signals (Mu et al. 2019). In the context of memory, NE afferents increase the frequency of miniature excitatory postsynaptic currents (mEPSCs) in the DG, a process that depends on astrocytic glutamate secretion (Shen et al. 2021). When pharmacologically blocking α_1_ARs, thus silencing the effect of NE afferents, contextual fear memory formation and short‐term consolidation are impaired (Shen et al. 2021). There are also noradrenergic projections from the LC into the anterior cingulate cortex (ACC), which have been shown to modulate pain‐related associative learning and avoidance behaviors through astrocytic β2‐adrenergic receptors (β_2_ARs) (Iqbal et al. 2023). While the ablation of LC noradrenergic neurons impairs visceral aversive memory without affecting acute pain behaviors, and the inhibition of ACC β_2_ARs disrupts aversive memory formation in the conditioned place avoidance (CPA) paradigm, optogenetic activation of astrocytic β_2_ARs in the ACC promotes aversive memory (Iqbal et al. 2023). Knocking down these receptors, specifically in ACC astrocytes, suppresses aversive learning without affecting spatial memory or anxiety‐related behaviors (Iqbal et al. 2023). Silencing astrocytic A_2A_ receptors in the CA1 decreases hippocampus‐dependent memory performance and impairs synaptic plasticity (Madeira et al. 2023). Together, these findings highlight the central role of astrocytic secretion of D‐serine and glutamate, and adrenergic receptor signaling, mediated by channels and receptors such as best1, CB1R, connexin30, and A2AR, in modulating region‐specific memory processes.

Astrocytes possess fine processes, known as peripheral astrocytic processes (PAPs) or “leaflets”, which are intimately involved in synaptic function. These structures are responsible for clearing neurotransmitters (e.g., glutamate) from the synapse, limiting their diffusion, and regulating extracellular ionic (e.g., potassium) concentrations (Armbruster et al. 2022). Using genetically encoded voltage indicators, several researchers measured rapid and localized depolarizations in PAPs driven by elevated extracellular potassium and glutamate during neuronal activity. These depolarizations inhibit astrocytic glutamate clearance and increase neuronal glutamate activation (Armbruster et al. 2022). The astrocytic leaflets may undergo structural plasticity, which has been linked to memory formation (see ‘Astrocytic effects on memory via plasticity’ section) (Badia‐Soteras et al. 2023). Contextual fear conditioning (FC)‐induced retraction of leaflets from hippocampal neuronal synapses is associated with increased extrasynaptic glutamate diffusion and fear memory expression (Badia‐Soteras et al. 2023). Memory deficits may also be rescued by a combined injection of D‐serine and glutamate when D‐serine seems to also reverse memory impairments induced by pharmacological inhibition of the astrocytic synthesis of the amino acid glutamine (Linsambarth et al. 2022). A molecule called l‐α‐aminoadipate inhibits glutamine synthetase, an enzyme that turns glutamate into glutamine, thus affecting glutamate levels in astrocytes. This molecule not only decreases NOR and long‐term potentiation magnitude in the CA1 but also alters astrocyte morphology, increasing the length and complexity of their processes (Pereira et al. 2021). Importantly, although this molecule is commonly referred to as a gliotoxin and is employed to impair astrocytic function, this classification should be approached with caution. Its effects are not necessarily specific to astrocytes and may extend to other cell types (Pereira et al. 2021). Moreover, even alterations that are specific to astrocytes, such as changes in astrocyte morphology and synaptic plasticity, can have broader implications that influence neuronal signaling. As such, interpretations of findings derived from its use must consider these broader impacts to avoid potential misattribution or oversimplification of its effects.

An important astrocytic protein localized in PAPs (specifically surrounding excitatory synapses) in the hippocampal CA1 region is the membrane protein MLC1 (Kater et al. 2023). Loss of MLC1 leads to structural changes in PAPs, including shortened tips and reduced spine contact. While the excitatory synaptic transmission is minimally affected in Mlc‐null mice, glutamate dynamics are altered in these mice, correlating with slower clearance of extracellular glutamate (Kater et al. 2023). This results in impaired contextual fear memory, suggesting a role for MLC1 in its formation or consolidation (Kater et al. 2023).

Interestingly, astrocytes may exhibit distinct Ca^2+^ activity patterns in response to different neurotransmitters, as suggested by conducting a two‐photon imaging of cortical slices bathed in either glutamate or GABA_B_ agonists. This work revealed that the glutamatergic agonist triggers robust, transient Ca^2+^ increases, with widespread, earlier‐onset propagating events, compared to the GABAergic one, which causes delayed and more prolonged activation (Cahill et al. 2024). A secondary messenger relevant to the astrocytic role in memory is inositol 1,4,5‐trisphosphate (IP3), which affects signal transduction in cells and mediates Ca^2+^ release from the endoplasmic reticulum. Its type 2 receptor (IP3R2) is the main isoform expressed by astrocytes, and knocking out IP3R2 disrupts Ca^2+^ signaling in these cells and impairs remote object recognition, contextual and cued fear memory, and spatial memory (Pinto‐Duarte et al. 2019).

Another study that investigated the interaction between GABA and astrocytes in the context of memory created transgenic mice with ablated astrocytic GABA_B_ receptors (GABA_B_Rs) in the medial prefrontal cortex (mPFC) (Mederos et al. 2021). This ablation impairs the working memory of mice in the alternating T‐maze test. This astrocytic manipulation also affects neurons, showing increased firing rates of both excitatory and inhibitory neurons during the T‐maze test and decreased gamma oscillation power, an oscillation considered a correlate with working memory (Buzsáki and Wang 2012). Some specific astrocytic populations, like Crym+ astrocytes in the striatum, can regulate neurotransmitter release probability through tonic GABA‐mediated presynaptic modulation (Ollivier et al. 2024). Knocking out Crym in mice leads to an impairment in novel object recognition (Ollivier et al. 2024).

To further our understanding of the bidirectional functional relationship between astrocytes and neurotransmitter signaling, researchers have developed many innovative techniques. One such method is Cell‐based Neurotransmitter Fluorescent Engineered Reporters (CNiFERs) for optical detection of neurotransmitters in vivo (Lacin et al. 2016). This technique allows for the detection of a wide range of neurotransmitters and neuromodulators that act through G protein‐coupled receptors (GPCRs) and provides valuable insights into neurotransmitter dynamics in the context of astrocyte function and memory processes (Lacin et al. 2016). For example, conditioned place preference (CPP) requires both reward‐based motivation and the creation of contextual memory. The activation of astrocytic μ‐opioid receptors is sufficient and necessary for CPP (Nam et al. 2019). These effects are dependent on glutamate released from astrocytes and the activation of presynaptic mGLuR, as observed by using iGluSnFR (Nam et al. 2019).

Astrocytic responses related to fear‐learning in mice are also mediated by nicotinic ACh receptors (nAChRs), which are required for memory persistence (Zhang et al. 2021). A footshock activates astrocytes via α7‐nicotinic acetylcholine receptors, then after mice learn the cued FC, there is a de‐novo, sound‐evoked astrocytic Ca^2+^ event. This event is present while the memory persists and disappears with extinction. Interestingly, knocking out the nAChRs impairs conditioned fear memory persistence.

Another type of cholinergic receptors is the muscarinic family of receptors. The muscarinic cholinergic receptor in the astrocytes of the DG plays a critical role in regulating adult hippocampal neurogenesis, and knocking out these receptors in DG astrocytes impairs conditioned fear memory (Li et al. 2022).

Altogether, these findings illustrate how the structure of astrocytes and astrocytic regulation of neurotransmission (including glutamate, GABA, D‐serine, NE, and Ach), and various receptors and signaling pathways (including NMDARs, GPCRs, and adrenergic, cannabinoid, and purinergic signaling), contributes to region‐specific memory processes.

## 3 The Role Interactions Between Astrocytes and Various Biomolecules Play in Memory

Recent research has shed light on the crucial role of astrocytes in memory formation and consolidation, highlighting the intricate interplay between these glial cells and various biomolecules. Astrocytes contribute to memory processes through multiple mechanisms, including metabolic support, stress response, neuro and glio‐modulation, and mitochondrial function.

### 3.1 Lactate and Glycogen Metabolism

Glycogen serves as the primary energy reserve in the brain. It is stored almost exclusively by astrocytes, where it can be rapidly broken down to generate metabolic substrates (i.e., lactate) as energy sources for neurons to support their function (Alberini et al. 2018). Lactate, as well as the glycogen‐metabolism processes that generate it in astrocytes, are repeatedly shown to be crucial to their role in memory. The selective inhibition of glycogen metabolism in astrocytes impairs learning in various memory‐related tasks, including NOR (Vezzoli et al. 2020), inhibitory avoidance (Descalzi et al. 2019), and METH‐induced CPP (Tan et al. 2022).

Interestingly, intra‐hippocampal administration of l‐lactate rescues memory deficits and reverses the plasticity changes (Vezzoli et al. 2020). L‐lactate is released from ACC astrocytes during aversive memory formation (Iqbal et al. 2022). Another approach to studying astrocytic function involves selectively activating these cells using chemogenetic or optogenetic techniques. For example, the Designer Receptor Exclusively Activated by Designer Drug (DREADD) hM3Dq, which induces elevation of Ca^2+^ levels in astrocytes through the Gq pathway (Adamsky et al. 2018). Inhibiting glycogen phosphorylation (glycogenolysis) by DAB administration or activating a DREADD with a Gi‐alpha unit (hM4Di) in ACC astrocytes by administering clozapine N‐oxide (CNO) leads to weaker aversive memory that can be rescued by exogenous lactate. The infusion of exogenous lactate also induces the expression of the memory‐related plasticity genes, which may explain the memory improvement (Iqbal et al. 2022). Other glycolytic metabolites downstream of lactate, such as pyruvate or the ketone body β‐hydroxybutyrate (B3HB) (Descalzi et al. 2019), are also able to rescue memory deficits due to the lack of learning‐induced astrocytic lactate production (Kambe et al. 2022).

When preferentially inhibiting metabolic functions in astrocytes using fluorocitrate, a transient astroglial metabolic inhibitor, in the basolateral amygdala (BLA) before contextual FC training or during the early stages of consolidation, memory is impaired. However, administration during recall or reconsolidation does not lead to that impact (Gargiulo et al. 2024). In addition, astrocytic monocarboxylate transporters (MCTs) play a crucial role in lactate shuttling. A temporally restricted knockdown of MCT1 and MCT4 (which are predominantly expressed in astrocytes) impairs long‐term memory formation, while knockdown of the neuronal MCT2 does not affect memory (Descalzi et al. 2019). These results suggest that astrocyte‐derived lactate is essential for memory consolidation.

### 3.2 Glucocorticoids

Glucocorticoids are adrenal steroid hormones that affect astrocytes through glucocorticoid receptors (GRs) that the astrocytes express. The complex relationship between astrocytic GRs and various aspects of memory, particularly in the context of fear and aversive memories, has also been explored recently. Mice that lack GRs specifically in astrocytes exhibit increased freezing behavior during non‐reinforced CS+ presentations in novel contexts after an auditory FC, displaying disrupted normal recall while fear extinction is unaffected (Taylor et al. 2021). The contextual fear memory in the CPA paradigm is impaired in transgenic mice without astrocytic GRs, but morphine‐induced CPP is not affected, suggesting a specific role of astrocytic GRs in aversive memory formation (Tertil et al. 2018).

Since astrocytes are known to provide metabolic support for neurons in memory formation and glucocorticoids are powerful modulators of metabolic processes, the effect of glucocorticoids on the metabolic status of astrocytes in vivo is a topic of interest. Researchers found that the induction of some metabolically relevant genes, specifically SGK1, is abolished when knocking out the glucocorticoids in astrocytes (Tertil et al. 2018). SGK1 was identified as a regulator of glucose uptake in astrocytes, and when knocking out glucocorticoids in astrocytes, it impairs aversive and fear memories (Tertil et al. 2018).

A brain region that is highly associated with fear‐related behaviors is the amygdala. When knocking down glucocorticoid receptors specifically in central amygdala (CeA) astrocytes, freezing time is increased (indicating an impaired memory) after FC (Wiktorowska et al. 2021), thus the connection between astrocytic GRs and aversive memory as seen in (Tertil et al. 2018) can also be demonstrated in the amygdala. With that, the manipulation of astrocytic glucocorticoid receptors in this brain region does not seem to affect performance in non‐aversive tests such as the NOR test (Wiktorowska et al. 2021). These results suggest that astrocytic glucocorticoids promote aversive memory consolidation and recall.

### 3.3 Adenosine and ATP

When astrocytes are stimulated, they can release ATP, which is broken down into adenosine in the extracellular space that then interacts with adenosine receptors (Hines and Haydon 2014). Recent studies explored the mechanisms by which astrocytic ATP/adenosine signaling modulates memory processes.

Optogenetic stimulation of Channelrhodopsin‐2 (ChR2) does not mimic the natural physiological activity in astrocytes. This type of stimulation in CA1 astrocytes leads to a decreased amount of freezing in a contextual FC test (Li et al. 2020), the number of alternations in the Y‐maze (Kim et al. 2024), latency in finding the correct well in the Barnes maze, and latency in entering the dark chamber in the passive avoidance test (Kim et al. 2024). The decreased memory expression in the contextual FC occurs only when the stimulation occurs during a specific time window of 1 h after training, but it is enough to induce a long‐term effect. This astrocytic‐ChR2 activation induces an increased concentration of extracellular ATP which in turn is degraded into adenosine. A1‐receptors (A1Rs) seem to mediate the contextual fear memory attenuation, demonstrated by the rescue of the fear memory when injecting an A1R‐antagonist within the first hour after training (Li et al. 2020).

Further insights into the role of astrocytic ATP/adenosine signaling in memory modulation come from a study done on the CeA, a key region in fear memory processing (Martin‐Fernandez et al. 2017). By chemogenetically activating astrocytic Gq‐DREADD, they showed that activated astrocytes, through the release of ATP/adenosine, depress excitatory synapses from the BLA via adenosine A1 receptor activation while enhancing inhibitory synapses from the lateral subdivision of the CeA through adenosine A2A receptor activation (Martin‐Fernandez et al. 2017). The physiological effect caused by the chemogenetic manipulation is accompanied by an impairment in contextual fear memory (Martin‐Fernandez et al. 2017). These results demonstrate how astrocytic ATP release and subsequent adenosine receptor activation can modulate memory expression in a region‐ and receptor‐specific manner.

### 3.4 Mitochondrial Function and Oxidative Stress

Recently, the connection between oxidative stress and mitochondrial functioning in astrocytes and memory is of rising interest. Older mice show cognitive decline and decreased performance in tasks such as the RAWM, accompanied by decreased expression of receptors for insulin‐like growth factor 1 (IGF1Rs) (Logan et al. 2018). Knocking down astrocytic IGFRs leads to decreased mitochondrial energy production, increased reactive oxygen species production, and impaired glucose and amyloid‐beta uptake. Importantly, IGFR deficiency results in more errors in the RAWM compared to control littermates (Logan et al. 2018). These findings suggest a strong association between age‐related decline in astrocytic IGFR signaling and impaired hippocampus‐dependent learning, highlighting the importance of astrocytic mitochondrial function in cognitive processes (Logan et al. 2018).

Neurodegenerative diseases, such as Alzheimer's disease, are closely associated with mitochondrial dysfunction, increased reactive oxygen species, neuroinflammation, and astrogliosis (Baier et al. 2022). SOD2, as mentioned before, is a mitochondrial protein relevant to memory‐related diseases. When knocking out SOD2 in hippocampal astrocytes, the oxidative stress marker 3‐NT is increased in male KOs but is decreased in the female KOs (while only male KOs show impaired learning and memory) (Baier et al. 2022). There seem to be no changes in morphology between astrocytes with and without the KO of the SOD2, but there is a significant decrease in the maximal astrocytic branch length in the male KO group compared with the male controls (Baier et al. 2022).

The studies covered in this section demonstrate how astrocyte‐derived metabolites and the interactions between astrocytes and various other biomolecules play a key role in modulating memory. Additionally, they demonstrate how different astrocytic proteins such as connexins, GPCRs, and mitochondrial proteins are essential for normal memory function, with their disruption leading to significant cognitive impairments.

## 4 Astrocytic Effects on Memory via Plasticity

Plasticity is the capacity of the nervous system to modify itself, functionally and structurally, usually in response to external experiences such as learning and creating memories (von Bernhardi et al. 2017). As mentioned before, an approach to studying astrocytic function and also their role in plasticity involves selectively activating these cells using chemogenetic or optogenetic techniques. The chemogenetic activation of the Gq pathway in CA1 astrocytes increases the frequency and the potency of spontaneous synaptic events and induces *de‐novo* plasticity, that is, it is sufficient to induce long‐term potentiation (LTP) at CA3‐CA1 synapses, even in the absence of any direct neuronal stimulation protocol. This potentiation is NMDAR‐dependent, lasts even when the astrocytic activation stops, and is mediated by D‐serine (Adamsky et al. 2018), supporting the previous literature which described that LTP depends on the release of D‐serine from astrocytes (Henneberger et al. 2010). Behaviorally, this manipulation enhances performance in the T‐maze paradigm and in the acquisition of contextual (but not cued) FC in mice (Adamsky et al. 2018; Refaeli et al. 2024). Importantly, the chemogenetic activation of neurons, instead of astrocytes, with the same type of DREADD leads to an impairment in memory performance because the astrocytes show an activity‐dependent effect (only on neurons active while creating the memory, not in home‐caged mice), whereas the neurons do not, so the active neurons mask the influence of the engram ones (Adamsky et al. 2018).

The effects of a chemogenetic activation of the astrocytic Gq pathways on memory and plasticity also occur in the infralimbic PFC, where the astrocytic activation also leads to improved performance in a NOR task and facilitates synaptic plasticity (Delcourte et al. 2023). On the other hand, chronically activating the Gq DREADD in ventral hippocampus astrocytes for 9 months leads to a decrease in average freezing during the acquisition of conditioned fear memory and an increase in recall (Suthard, Jellinger, et al. 2023).

The research techniques available to manipulate GPCRs in astrocytes are continuously developed. For example, even though astrocytes respond to many NTs via GPCRs (Durkee et al. 2019), there was no method to attenuate astrocytic GPCR activity in vivo until recently. iβARK (122 residue inhibitory peptide from β‐adrenergic receptor kinase) attenuates Gq‐dependent Ca^2+^ signaling in brain slices and in vivo, reduces startle‐evoked astrocytic Ca^2+^ when an air puff is given, or blocks the hyperactivity induced in an open field (OF) paradigm by the chemogenetic activation of a Gq DREADD in the striatum (Nagai et al. 2021). Importantly, iβARK does not affect the morphology or the spontaneous activity of striatum astrocytes (Nagai et al. 2021). This inhibitor and other future tools will contribute to our understanding of the effects astrocytic Ca^2+^ events have on neuronal activity and behavior.

The Gq pathway can also be activated optogenetically. For example, melanopsin is a light‐sensitive G‐protein coupled photopigment that can be expressed specifically in astrocytes to trigger G‐protein‐dependent Ca^2+^ signaling in them (Mederos et al. 2019). The optostimulation of melanopsin induces an increased excitatory synaptic transmission in CA1 neurons and drives hippocampal long‐term synaptic plasticity. Another optogenetic Gq‐GPCR construct that can activate the Gq pathway is Opto‐α1AR. When expressed in ACC astrocytes, brief light stimulation creates astrocytic Ca^2+^ events and suppresses neuronal activity in layers 2 and 3 of the somatosensory cortex (Iwai et al. 2021). The studies that optogenetically activated the Gq pathway in astrocytes demonstrate how the activation of astrocytes by photostimulation induces plasticity and, importantly, improves memory performance in a novel object location (NOL) task (Mederos et al. 2019), NOR (Iwai et al. 2021), and contextual FC (Adamsky et al. 2018). These findings highlight the ability of astrocytes to independently induce synaptic plasticity and influence memory formation.

Another α‐unit of GPCRs can be Gs. The chemogenetic activation of the Gs‐pathway in mPFC astrocytes during encoding or consolidation of conditioned fear memory does not influence remote fear memory expression, formation of cortical engram ensembles, or the reactivation of the engram during recall (Orr et al. 2015). These results suggest that increased Gs‐coupled receptor signaling in cortical astrocytes may not play a critical role in the formation of persistent cortical memory engrams. With that, the Gs‐coupled adenosine2A receptor is more abundant in humans with Alzheimer's disease and in aging mice that express human amyloid precursor protein, and their ablation increases levels of Arc (an immediate early gene required for long‐term memory) and improves memory performance (Orr et al. 2015). This highlights potential differences in astrocytic GPCR signaling effects based on brain region, timing of manipulation, and specific G‐protein pathways involved (Mak et al. 2024).

As mentioned before, an alternative pathway that can be chemogenetically activated is the Gi pathway, by the chemogenetic activation of the hM4Di DREADD. Activating this DREADD in astrocytes in the CA1 during the acquisition of FC impairs remote (but not recent) contextual fear memory (Kol et al. 2020), by preventing the recruitment of the ACC during memory acquisition via specific inhibition of the recruitment of neurons in the CA1 that project to the ACC. Notably, the inhibition of the CA1 → ACC communication during learning impairs the acquisition of remote, but not recent memory (Kol et al. 2020). These results suggest that astrocytes influence memory consolidation by modulating the communication between hippocampal and cortical regions and demonstrate a novel capacity of astrocytes to affect their neighboring neurons based on their projection target.

As mentioned before, the ACC also receives input projected from the LC. Optostimulation of LC neurons enhances aversive memory‐related behaviors and promotes plasticity‐related gene expression (Iqbal et al. 2023), although the combination of opto‐stimulation of the LC neurons along with chemogenetic activation of the astrocytic Gi‐pathway in the ACC counteracts this effect through the change in astrocytic activity, suppressing both the aversive memory and the associated plasticity‐related gene expression (Iqbal et al. 2023). Chemogenetic activation of this Gi‐pathway in striatal astrocytes leads to significant impairment in NOR memory (Nagai et al. 2019), and it induces LTP in CA1 astrocytes which accounts for the contextual memory acquisition required to induce CPP (Nam et al. 2019).

G‐protein coupled receptor 30 (GPR30) is a GPCR, which its activation decreases cyclic AMP (cAMP; a secondary messenger important to many biological processes) levels in the cell, similarly to the Gi pathway. It has a critical role in astrocytic involvement in learning and memory processes in female mice (Wang et al. 2023). An astrocyte‐specific deletion of GPR30, but not neuron‐specific deletion, leads to impaired performance in NOR tasks, decreased LTP in neurons, and a simplified astrocytic morphology. A targeted deletion of GPR30 in CA1 astrocytes results in impaired memory in both NOR and contextual FC tasks. Importantly, this deletion alters the balance between two astrocytic phenotypes: the A1 phenotype, which is referred to as an inflammatory phenotype as it expresses more genes that destroy synapses, and the A2 phenotype, which is referred to as protective as it expresses many neurotrophic factors. Specifically, GPR30 deletion in CA1 astrocytes leads to an upregulation of the A1 phenotype and a downregulation of the A2 phenotype. The restoration of GPR30 expression in astrocytes rescues the observed functional impairments. Mechanistically, this effect is mediated by PJA1 via CREB signaling, particularly through Serpina3n, which is involved in anti‐inflammatory responses (Wang et al. 2023).

A different approach to investigating astrocytic involvement in memory is via an increase in cAMP levels in astrocytes and observing the effects on different types of memory stages, as was done in a transgenic mouse line using light stimulation (Zhou et al. 2021). This study showed that increasing cAMP levels in hippocampal astrocytes during memory formation improves NOL memory, but increasing it during memory retention impairs memory. These effects, and also the induction of neuronal activity and plasticity (increase in fEPSP amplitude) following light stimulation, are mediated by NMDARs and lactate shuttling (Zhou et al. 2021).

The importance of astrocytic signaling in memory processes and plasticity is further supported by studies manipulating specific astrocytic proteins. For instance, connexin43 is a membrane protein that also forms gap‐junction channels between astrocytes. Blocking astrocytic connexin43 hemichannels in the BLA decreases post‐synaptic NMDAR‐dependent currents and impairs short‐term cued fear memory (Linsambarth et al. 2022). Blocking connexin43 in the PFC leads to impeded working memory in a delayed alternation task (Tao et al. 2021). Upregulating the expression of connexin30 in CA1 astrocytes increases the connectivity of the astrocytic network in this area but decreases the spontaneous and evoked synaptic transmission in neurons. Alongside the reduced neuronal excitability, NOR memory is also impaired (Hardy et al. 2023).

Additionally, knocking out both connexin30 and connexin43 leads to morphological changes in astrocytes and microglia. In addition, a pro‐inflammatory‐like activation of both alters synaptic transmission and Ca^2+^ activity in hippocampal astrocytes and impairs long‐term spatial learning in the Barnes maze (Hösli et al. 2022). Notably, connexin43 hemichannels are key mediators of glutamine release, and astroglial glutamine supply via these hemichannels sustains glutamatergic synaptic transmission and is required for NOR memory (Cheung et al. 2022). Other morphological changes in astrocytes may occur following depletion of certain morphology‐related genes (ermt2 or Ezr) using CRISPR/Cas9. The genetic ablation of each of these genes in hippocampal astrocytes leads to impaired memory in the NOL paradigm (Endo et al. 2022).

Furthermore, astrocytes play a role in regulating synapse formation and elimination, which can impact memory processes. Ephrin‐B1 is a membrane‐bound protein that acts as a ligand for the EphB receptors, and the interaction between these proteins in neurons is important in the formation and maintenance of synapses. The astrocytic ephrinB1 acts as a negative regulator of synaptogenesis; therefore, knocking it out enhances fear memory, and over‐expressing it impairs contextual memory (Koeppen et al. 2018). It also impairs astrocytic engulfment ability and restricts new synapse formation in the hippocampus (Koeppen et al. 2018). When ephrin‐B2 (another member of the membrane‐bound family) is depleted in BLA astrocytes, both contextual and auditory‐cued fear memory are impaired (Agarwal et al. 2024).

Memory is also impaired by the conditional KO of the transcription factor nuclear factor I‐A (NFIA) in astrocytes (Huang et al. 2020). This KO leads to a decreased morphological complexity and reduced Ca^2+^ events frequency and amplitude in hippocampal astrocytes. Thus, it impairs their interactions with neurons and inhibits their synaptic plasticity, which manifests in impaired contextual fear memory and NOR (Huang et al. 2020).

The structural plasticity of astrocytic distal processes (“leaflets”) has been linked to memory formation. As mentioned in the section discussing neurotransmitters, contextual FC induces a transient retraction of astrocyte leaflets from hippocampal synapses (persisting for days before returning to normal) and enhances fear memory expression (Badia‐Soteras et al. 2023). This retraction is also associated with enhanced activation of NMDA receptors leading to increased neuronal activation (Badia‐Soteras et al. 2023). NMDA receptor activity is affected by the release of D‐serine and glutamate from astrocytes in the CA1. This co‐release is the product of input into the CA1 from the CA3 and the LC (Koh et al. 2022). The change in NMDAR activity increases synaptic plasticity, particularly during spatial memory acquisition. This results in heightened long‐term depression (LTD) and metaplasticity, with increased heterosynaptic LTD influenced by high‐frequency stimulation in CA3 that raises NE levels. This process is dependent upon α1‐adrenergic receptors (Koh et al. 2022). Importantly, increased activation of NMDARs can also be observed following an astrocyte‐specific depletion of ezrin (a structure‐related gene) using CRISPR/cas9 (Badia‐Soteras et al. 2023). This manipulation also results in shorter astrocyte leaflets, reduces morphological complexity, and reduces astrocytic contact with the synaptic cleft. Moreover, after FC, these cellular phenotypes translate to increased recall‐evoked activation of CA1 pyramidal neurons and enhance fear memory expression (Badia‐Soteras et al. 2023).

The role of astrocytes in regulating synapse formation and elimination is demonstrated by the astrocytic phagocytosis of synapses in an activity‐dependent manner, particularly targeting excitatory synapses in the CA1 region which affects NOR and NOL memory (Lee et al. 2021). Astrocytes regulate synapse formation and elimination by proteasomic activity which degrades proteins as well. When using astrocyte‐specific CRISPR to inhibit proteasome‐mediated protein degradation in the amygdala, fear memory in contextual testing is impaired (Farrell et al. 2023). These studies demonstrate the crucial role of astrocytic morphology in memory formation through their interactions with synaptic activity and plasticity.

Altogether, in this section, astrocytes are shown to contribute to synaptic plasticity through various mechanisms and exhibit functional diversity across different areas of the brain. These findings highlight the necessity for region‐specific studies to gain a comprehensive understanding of their role in memory. Additionally, this section demonstrated how the use of optogenetics and chemogenetics has greatly improved our capability to investigate astrocyte activity in memory‐related processes, leading to fresh insights into their signaling functions and role in different stages of memory.

## 5 The Future Is Now. Recent Methods That Are Increasingly Employed in the Study of Astrocytes in Memory

### 5.1 Ca^2+^ Imaging in Astrocytes as a Tool for Investigating Astrocytic Involvement in Memory

Ca^2+^ transients in astrocytes have been widely used as a proxy for astrocyte activity and signaling (Bazargani and Attwell 2016). Ca^2+^ imaging is possible at the population level (e.g., bulk imaging using fiber photometry) or at a single‐cell resolution (e.g., with two‐photon microscopy). When imaging BLA astrocytes using photometry during contextual FC, recall, and extinction, astrocytic Ca^2+^ responses tuned to the initiation and termination of freezing bouts during memory recall, but not during extinction, are observed (Suthard, Senne, et al. 2023).

The measurement of population Ca^2+^ activity in hippocampal astrocytes reveals changes that occur during the encoding, maintenance, and retrieval of working memory as well. Fluorescent traces show three peaks just before the reward is received, and a reduced astrocytic Ca^2+^ signal is observed during retrieval after rule learning (Lin et al. 2022). In this study, neuronal and astrocytic activities in the hippocampus appear to be interlocked in an antagonistic relationship, that is, heightened astrocytic activity is following increased neuronal activity and constraining it until the reward is received (Lin et al. 2022). This relationship is accompanied by enhanced synchronization of task‐responsive astrocytic Ca^2+^ signals between the hippocampus, medial PFC, and the striatum (Lin et al. 2022).

Imaging astrocytic Ca^2+^ activity at a single‐cell resolution is challenging, yet several laboratories have already succeeded at this task (Curreli et al. 2022; Doron et al. 2022; Rupprecht et al. 2024). Learning in mice can be tested by chronic two‐photon imaging of CA1 astrocytes as mice run in familiar and novel virtual reality environments to obtain water rewards. This study revealed a ramp in Ca^2+^ activity in these astrocytes approaching a reward, but only after learning the maze, and not in a novel environment in which the ramping activity disappears (Doron et al. 2022). These results indicate that astrocytes might encode the learned reward location in spatial contexts. In addition, recent findings suggest that astrocytes might be integrators of past events, rather than predictors of future rewards (Rupprecht et al. 2022). These findings extend the known computational abilities of astrocytes and their role in cognitive functions such as memory.

With the development of advanced fluorescent tagging and two‐photon microscopes that allow the animals to freely move in an experimental arena (Zong et al. 2022), we expect to see more studies that widen and deepen our knowledge of astrocytic activity during different stages of memory and the role of this activity in regulating memory.

### 5.2 Optogenetic and Chemogenetic Manipulations as Tools for Studying the Role of Astrocytes in Memory

In order to explore the role of astrocytes in memory processes more deeply, it is essential to be able to control their activity in a manner that is specific to both the cell type and the timing and location of the manipulation. Pharmacological manipulations involve the administration of drugs that can modulate the activity of particular types of receptors, ion channels, or signaling pathways, as indicated in many cases above. This type of manipulation provides a means to dissect the roles of different molecules and pathways in neural processes and their influence on behavior, which is an important time‐restricted manipulation, but it often lacks cell‐type specificity. In comparison, developing transgenic lines can be cell‐type specific, but are not temporally restricted, leaving room for the development of compensating mechanisms over time.

Optimal strategies that are both temporally accurate and cell‐type‐specific are opto−/chemo‐genetic manipulations, also described above. These methods employ light or drugs to modulate cellular activity, allowing the investigation of the effects of the activation or the inhibition of cells under otherwise unmanipulated physiological circumstances (Yu et al. 2020).

Employing methods such as chemo‐ or optogenetic manipulation of astrocyte activity during the stages of memory acquisition, consolidation, or recall provides fascinating insights into the involvement of astrocytes in memory processes and the interactions between astrocytic activity and neuronal responses. In the near future, the current toolbox will be enhanced by advanced opto‐ and chemo‐dependent designs of various constructs that will help deepen our understanding of the role of astrocytes in memory and their functions in general.

One example of such a study used modified RAC1 as an optogenetic tool. RAC1 is an important biomolecule, a GTPase involved in cytoskeletal reorganization and astrocyte morphology in memory processes (Fan et al. 2021). By reconstructing RAC1 as photo‐activatable, inactivatable, or insensitive, it can be observed that both up‐ and down‐regulation of RAC1 in BLA astrocytes decreases neuronal cFOS levels during the acquisition and retrieval of contextual and cued FC (Fan et al. 2021). This finding suggests that RAC1 affects memory in an inverted U‐shaped manner, where an optimal level is required for proper function, and any deviation from this level in either direction results in dysfunction.

These genetic tools are constantly being developed. Some of the developments try to improve the efficiency of genetic manipulation or cell‐type specificity, and some of them try to allow other functions other than elevating Ca^2+^ levels in the cells, like the iβARK and CalEX mentioned earlier. A recently published paper used in vivo optogenetic stimulation of NE afferents to the hippocampus of mice, and then also iβARK and CalEX in hippocampal slices to demonstrate how astrocytes, not neurons, are the primary mediators of NE's modulatory effect on synaptic transmission (Lefton et al. 2025). This paper, alongside two others that were conducted in zebrafish larvae (Chen et al. 2025), Drosophila flies, and rat astrocytic cultures (Guttenplan et al. 2025), described a preserved mechanism across species, in which NE signals cause elevations of Ca^2+^ in astrocytes, which leads to astrocytic release of ATP. When outside the astrocytes, this ATP is transformed into adenosine, which activates A1 adenosine receptors of neurons, eventually inhibiting neuronal synaptic transmission (Lefton et al. 2025) and behavioral changes (Chen et al. 2025). As mentioned in the section called “Astrocytic effects on neurotransmitters actualize their role in memory”, NE is one of the main inputs to the hippocampus, which raises intriguing questions about how these advancing tools can help us investigate the effects of the NE signaling pathway on memory encoding and recall in hippocampus‐dependent memory tasks, and maybe also investigate if astrocytic dysfunction in this pathway contributes to memory impairments in the pathologies that involve dysregulation of neuromodulators.

### 5.3 Investigating Engrams

Now that the field of astrocytes in memory research has established itself as a recognized area of study and significant evidence for the involvement of astrocytes in various stages of memory has been provided, it is possible to begin exploring questions regarding the existence of astrocytic engrams. Recent studies look at the dynamics of memory engrams, ensembles of cells throughout the brain that are formed during the acquisition of memory and are necessary for recall (Josselyn and Tonegawa 2020), and the role of astrocytes in their formation and maintenance. CA1 neuronal engrams remain stable between recent and remote recall, and inhibition of engrams for recent recall during remote recall functionally impairs memory (Refaeli et al. 2023). In addition, astrocytic activation can differentially affect recent and remote memory recall, specifically in their effect on CA1 → ACC projections (Refaeli et al. 2024). These findings suggest that astrocytes may play a role during consolidation that affects the selection of the engram neurons, which can be seen even 30 days later and raises the question of whether there are astrocytic engrams.

Two recent papers aimed to answer this question, paving the way for more future research of this exciting nature. In both works, the authors developed a novel genetic tool to label and manipulate specific subsets of astrocytes during a task. Williamson et al. did so in the hippocampus during fear conditioning, and Serra et al. in the nucleus accumbens (NAc) during operant conditioning. Williamson et al. found that learning events of FC activate specific ensembles of hippocampal astrocytes which are closely associated with neuronal engrams involved in memory, and exhibit distinct expression of different transcription factors and c‐Fos (Williamson et al. 2025). In addition, they demonstrated how the selective Gq‐pathway chemogenetic reactivation in these astrocytic ensembles is sufficient to trigger fear‐memory recall (Williamson et al. 2025), which strengthens the claim that they regulate the activity of neuronal engrams and are involved in memory. In the work by Serra et al., the astrocytic ensembles were labeled during an operant conditioning task near one of two ports. The reactivation of these ensembles biases the behavior of the mice, leading them to preferentially choose the port associated with the original tagging (Serra et al. 2025).

These studies highlight the potential of developing innovative techniques to unravel the role of astrocytes in memory, particularly in the context of engram formation and maintenance. As our understanding of astrocytic contributions to memory and cognition expands, it is reasonable to anticipate that our field of research will witness an increasing number of studies utilizing advanced techniques to explore astrocytic engrams with greater precision and depth.

## 6 Conclusions

The studies summarized in this review highlight the critical role of astrocytes in memory processes, revealing their complex interactions with neurotransmitters, various other biomolecules, and different neural circuits. These studies highlight the importance of expanding the conceptual models when investigating neural circuits, as they demonstrate how astrocytes are active participants in neurotransmission, which can affect memory formation. They respond to and modulate various neurotransmitters, including glutamate, GABA, norepinephrine, and acetylcholine, through specific receptors and signaling pathways.

Calcium signaling in astrocytes is a crucial mechanism for their involvement in memory processes, and Ca^2+^ imaging techniques have revealed distinct activity patterns in astrocytes during different phases of memory formation, consolidation, and recall. These Ca^2+^ patterns do not merely mirror the neuronal activity; they indicate that astrocytes integrate and regulate it, sometimes in a projection‐specific or memory task‐dependent manner. Moreover, astrocyte‐derived metabolites, particularly lactate, play a vital role in memory consolidation. The inhibition of astrocytic glycogen metabolism or lactate transport impairs various forms of memory. The interaction between astrocytes and various other biomolecules, such as glucocorticoids, ATP, and adenosine, is also shown to modulate memory processes in complex ways.

Astrocytic proteins, including connexins, GPCRs, and mitochondrial proteins, are crucial for normal memory function, as manipulations of these proteins can lead to significant alterations in memory performance. In addition, astrocytes are also shown to contribute to synaptic plasticity through multiple mechanisms, including the release of gliotransmitters, regulation of extracellular glutamate levels, and structural changes in their processes.

In conclusion, astrocytes exhibit remarkable heterogeneity in their functions across different brain regions, highlighting the need for region‐specific studies to fully understand their role in memory. In addition, techniques such as optogenetics, chemogenetics, and high‐resolution imaging have greatly enhanced our ability to study astrocyte function in memory processes, revealing new insights into their signaling capabilities. With emerging tools and technologies on the horizon, the field is set for even more exciting discoveries. The findings described in this review collectively demonstrate that astrocytes are not merely supportive cells but are integral components of the neural circuits underlying memory. They actively participate in information processing, synaptic plasticity, and memory formation through diverse mechanisms.

Future research should employ newly advanced tools to gain insights on the astrocytic role in high brain functions such as learning and memory, while focusing on clarifying the specific molecular mechanisms by which astrocytes influence different types of memory, investigating the role of astrocyte heterogeneity in memory processes across various brain regions, and developing more precise tools for manipulating astrocyte function in vivo. Moreover, it is important to keep exploring the potential of astrocyte‐targeted interventions for treating memory disorders.

As our understanding of astrocyte function in memory continues to grow, it may lead to new therapeutic approaches for cognitive disorders and a more comprehensive view of how the brain processes and stores information.

## Author Contributions

**Shay Meron Asher:** conceptualization, data curation, formal analysis, visualization, writing – original draft, writing – review and editing. **Inbal Goshen:** conceptualization, supervision, writing – original draft, writing – review and editing.

## Consent

Informed consent was achieved for all subjects, and the experiments were approved by the local ethics committee.

## Conflicts of Interest

The authors declare no conflicts of interest.

## Data Availability

The authors have nothing to report.
